# Experimental and Density Functional Theory Studies on 1,1,1,4,4,4-Hexafluoro-2-Butene Pyrolysis

**DOI:** 10.3390/molecules25173799

**Published:** 2020-08-21

**Authors:** Neng Tao, Changcheng Liu, Haoran Xing, Song Lu, Siuming Lo, Heping Zhang

**Affiliations:** 1 State Key Laboratory of Fire Science, University of Science and Technology of China, Hefei 230027, China; taoneng@mail.ustc.edu.cn (N.T.); ccliu2017@ustc.edu.cn (C.L.); xinghr@mail.ustc.edu.cn (H.X.); lusong@ustc.edu.cn (S.L.); 2 Department of Civil and Architectural Engineering, City University of Hong Kong, Tat Chee Avenue, Kowloon 999077, Hong Kong; sm.lo@cityu.edu.hk

**Keywords:** HFO-1336mzz(Z), thermal decomposition, DFT, GC–MS

## Abstract

A series of thermal decomposition experiments were conducted over a temperature range of 873–1073 K to evaluate the thermal stability of 1,1,1,4,4,4-hexafluoro-2-butene (HFO-1336mzz(Z)) and the production of hydrogen fluoride (HF). According to the detected products and experimental phenomena, the thermal decomposition of HFO-1336mzz(Z) could be divided into three stages. Our experimental results showed that HF concentration gradually increased with the elevation of thermal decomposition temperature. In this present study, a total of seven chemical reaction pathways of HFO-1336mzz(Z) pyrolysis were proposed to explore the generated mechanism on products through density functional theory (DFT) with M06-2X/6-311++(d,p) level theory. The thermal decomposition mechanism of pure HFO-1336mzz(Z) was discussed and the possible formation pathways of HF and other main products were proposed.

## 1. Introduction

Due to the limitations for the application of hydrofluorocarbons (HFCs) with high global warming potential (GWP), the working fluid with high GWP, for instance, HFC-245fa with a GWP of 858 would be replaced. In the past decades, the working fluid with low global warming potential has attracted great attention [1]. HFO-1336mzz(Z) (*cis*-CF_3_CHCHCF_3_; 1,1,1,4,4,4-hexafluoro-2-butene; GWP = 2, ODP = 0; previously referred to as DR-2) is a kind of clear, colorless, lower toxic and non-combustible liquid (safety classification: A_1_) [2,3]. As a novel type of low GWP working fluid with high-temperature chemical stability, HFO-1336mzz(Z) not only benefited the natural environment, but it can also enable 17% higher net cycle efficiencies over HFC-245fa [4,5,6]. Moreover, HFO-1336mzz(Z) demonstrated higher thermal performance than HCFO-1233zd (E) in the organic Rankine cycle (ORC) and vapor compression cycle systems when it was used as a kind of ORC working fluid [7]. To date, HFO-1336mzz(Z) was also used as a foam-blowing agent, refrigerant, solvent and fire extinguishing. HFO-1336mzz(Z) was studied as fire extinguishing and considered to have the great potential to be a promising environment-friendly halon alternative [8,9]. However, HF would be produced in the process of extinguishing the fire when HFO-1336mzz(Z) used as fire extinguishing. Wang et al. [8] also investigated the thermal decomposition properties of HFO-1336mzz(Z) with air over a temperature range of 373–1073 K and the products.

Some previous studies have focused on the properties of HFO-1336mzz(Z) and the thermal decomposition mechanism and useful information about HFO-1336mzz(Z) has been obtained. Huo et al. [10] studies the pressure (2.1, 3.1 and 4.0 MPa) effect on dissociation of HFO-1336mzz(Z) for 24 h and the dissociation temperatures are 583 K to 603 K, 563 K to 583 K and 543 K to 563 K. Huo et al. [11] studied the products and pyrolysis process of HFO-1336mzz(Z) at 2000–3000 K by using the ReaxFF simulation, demonstrating that the thermal decomposition of HFO-1336mzz(Z) was assigned into three stages and proposed the dissociation mechanism. The settings of high temperatures are favored in reactive MD simulations in order to promote sufficient atomic motion and molecular collisions, which can speed up the reaction and reduce the simulation time. Therefore, the experimental thermal decomposition temperature of HFO-1336mzz(Z) is lower than that of molecular dynamics simulation (2000–3000 K). This research team also studied the effects of Cu, O_2_, H_2_O and POE lubricant on the thermal decomposition of HFO-1336mzz(Z) [12,13,14,15]. Results showed that Cu could promote the dissociation and oxidation decomposition of HFO-1336mzz(Z) and produce HF, COF_2_ and CO_2_ at 2000–3000 K. Moreover, they experimental results also showed that the dissociation temperature range of HFO-1336mzz(Z) with lubricant at four megapascals was 523–543 K. Furthermore, Tanaka et al. [16,17] studied the thermodynamic properties of HFO-1336mzz(Z) and measured the critical parameters, such as density, temperature and pressure of HFO-1336mzz(Z) by using the meniscus disappearance method.

At present, the pyrolysis products of some HFCs have been studied in the thermal decomposition experiments. Hu et al. [18] investigated the 1,1,2,3,3,3-hexafluoro-propene (HFP) pyrolysis at various temperatures (673–1073 K) and times (3 s, 30 s), demonstrating that hydrogen fluoride (HF) elevates by increasing the temperature and time. Also, three cyclo-compounds were first discovered in the products of HFP pyrolysis. Zhou et al. [19] studied the thermal decomposition products of 2-bromo-3,3,3-trifluoropropene (2-BTP) and its formation mechanism by combining experimental data and theoretical calculation results using Gaussian 03. Ting et al. [20] performed density functional theory (DFT) calculations using Dmol^3^ code to reveal the C_2_HF_5_ pyrolysis mechanism and adopted three kinds of analysis methods to identify the decomposition products. Kontomaris [6] found that HFO-1336mzz(Z) was chemically stable for decomposition, polymerization, oxidation and hydrolysis. Moreover, HFO-1336mzz(Z) was also very stable under conditions of copper, carbon steel and aluminum at 523 K. However, the chemical stability of HFO-1336mzz(Z) at a higher temperature (higher than 523 K) has not yet been investigated.

Density functional theory (DFT), a most common computational quantum mechanical modeling method, has been widely applied to chemistry to investigate the chemical reactions of bond breaking and formation. Wang et al. used [9] density functional calculation to study some products of HFO-1336mzz(Z) further react with active OH• and H• radicals. Zhang et al. [21] explored the primary reaction pathways for CF_3_CF=CH_2_ decomposition and reported its thermal decomposition mechanism first, by using this method. Huang et al. [22] also used this method to investigate the primary products of thermal decomposition for lignin, including CO, CO_2_ and CH_4_ and their formation mechanisms. Of course, this DFT method has also been used to study the thermal decomposition for other organics and biomass [23,24,25,26,27,28].

To date, most related HFO-1336mzz(Z) pyrolysis studies were focused on the thermal decomposition mechanism. To obtain the products in the HFO-1336mzz(Z) thermal decomposition process more accurately and systematically, the characteristics of *cis*-CF_3_CH=CHCF_3_ pyrolysis were further explored experimentally and theoretically. In this work, the products and their formation mechanisms of HFO-1336mzz(Z) pyrolysis were presented at the molecule level, which provides a reference for reducing the yields of HF and other harmful macromolecular products and the induced impairs to human health and environment. Therefore, some studies needs to be focused on the generation quantity and formation mechanism of HF in HFO-1336mzz(Z) pyrolysis process.

## 2. Results and Discussion

### 2.1. Experimental Results and Discussion

#### 2.1.1. Thermal Decomposition Temperature and Products

**Figure 1:**
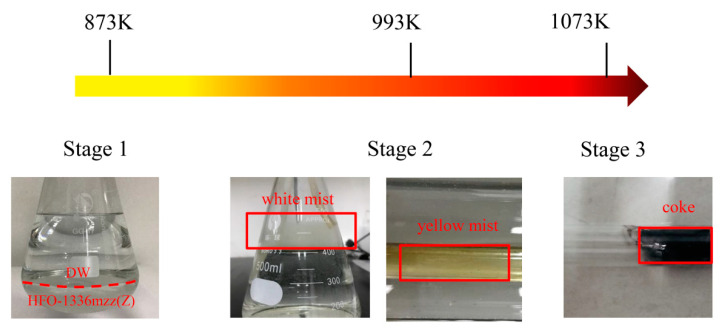
Crucial thermal decomposition temperature and phenomenon.

When the flow rate of the peristaltic pump was adjusted to 0.41 mL/min, stable bubbles were observed in the conical flask containing deionized water during pyrolysis. Figure 1 provides an overview of the HFO-1336mzz(Z) pyrolysis process at three stages. It was found that HFO-1336mzz(Z) slightly decomposed at 873 K, which was proved by the collected GC–MS products in a gas pocket and the pH and fluoride ion concentration in deionized water. Moreover, the phenomenon of delamination could be observed in deionized water and there was condensed HFO-1336mzz(Z) on the bottom of the deionized water. When the reaction temperature arrived at 973 K, the decomposition would become more intense. It is very interesting to find that the gas in the quartz tube would turn into a white mist, which was confirmed to be HF. Furthermore, the gas would turn into the yellow mist when the reaction temperature held at 993 K. As shown in Figure 1, when the reaction temperature arrived at 1073 K, coke rapidly forms, which shows that HFO-1336mzz(Z) not only perform simple pyrolysis, but also performed cyclization and polymerization reactions at high temperature.

#### 2.1.2. GC–MS Results

##### Gas Products

**Figure 2:**
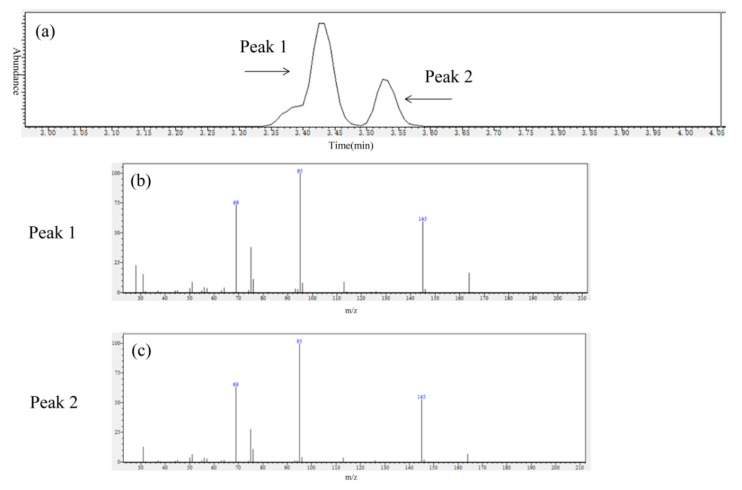
GC–MS results of gas collected by gas pocket. (**a**): Gas chromatogram of gas; (**b**): Mass spectrum of Peak 1; (**c**): Mass spectrum of Peak 2).

**Figure 3:**
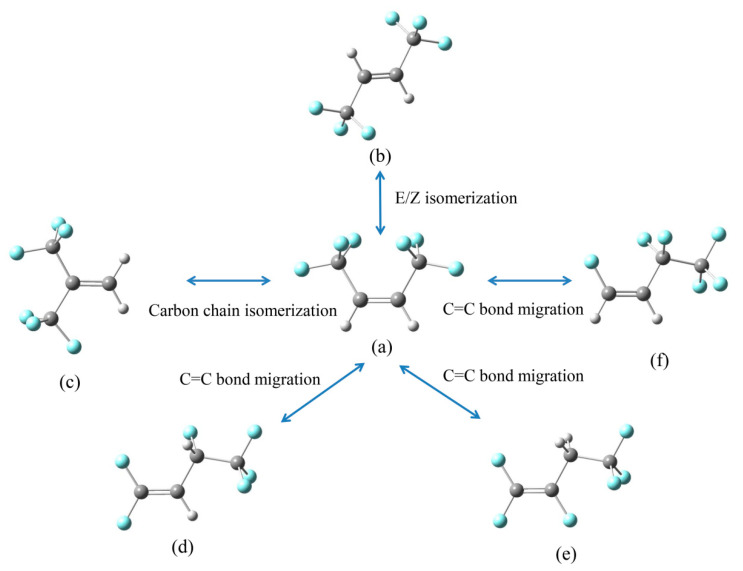
Possible isomerization reaction of HFO-1336mzz(Z). C, H and F atoms are represented with black, gray and blue balls, respectively (**a**): HFO-1336mzz(Z); (**b**–**f**): Possible isomerization of HFO-1336mzz(Z)).

GC–MS was used to identify the gaseous products of HFO-1336mzz(Z) pyrolysis. As shown in Figure 2a, GC–MS analysis revealed two main peaks and other small peaks needed to be further explored. The results of MS in Figure 2b,c suggest that there were HFO-1336mzz(Z) and its isomers. The possible isomeric molecular structures are shown in Figure 3. A recent work measured the HFO-1336mzz(E) concentration in glass tubes originally containing neat HFO-1336mzz(Z) after aging at 448 K and 473 K for 14 days in the presence of carbon steel, copper and aluminum [6]. However, the stereo isomerization of HFO-1336mzz(Z) to HFO-1336mzz(E) was extremely little, and the experimental results reported by Kontomaris shows that the concentrations of HFO-1336mzz-E were 0.004 and 0.011 at 448 K and 473 K. HFO-1336mzz(E) is a potential candidate for Halon Substitutes based on experimental and theoretical results of Zhang [29].This team found that The isomerization reaction of HFO-1336mzz(E) occurs after the environmental temperature increases to above 773 K, and HFO-1336mzz(Z) could be produced in this endothermal reaction. HFO-1336mzz(Z) and HFO-1336mzz(E) are cis–trans isomerism and this isomerization reaction was shown with the conversion between (a) and (b) in Figure 3. The van der Waals repulsion was relatively large since the two CF_3_ groups of the cis structure were relatively close to, causing that the stability of the cis structure was worse than that of the trans structure. Stereo isomerization of HFO-1336mzz(Z) to HFO-1336mzz (E) was thermodynamically favored due to the lower molecular energy of the E (or trans) isomer (about 5 kcal/mol). In other words, HFO-1336mzz(Z) under the high temperature conditions was likely to be converted to HFO-1336mzz(E).

In addition to the cis–trans isomerization reaction, it was also possible to carry out the double bond isomerization reaction and the carbon chain isomerization reactions. HFO-1336mzz(Z) would be converted to (c), (d), (e) and (f) via the carbon chain isomerization reaction and the double bond isomerization reaction when the thermal decomposition temperature reached up to 873 K, which needs to be further explored.

##### Oily Liquids Products

**Figure 4:**
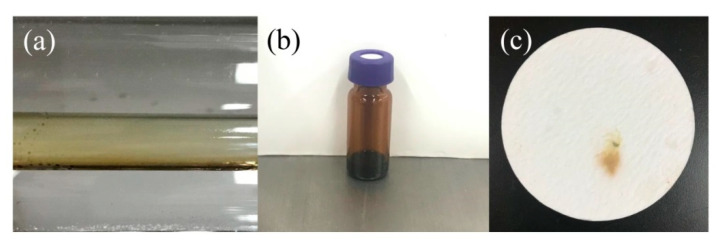
Flavescent oily liquids collected from the inner wall of the quartz tube: (**a**): oily liquids in the quartz tube; (**b**): collected from the quartz tube; (**c**): collected by cambridge pad.

**Figure 5:**
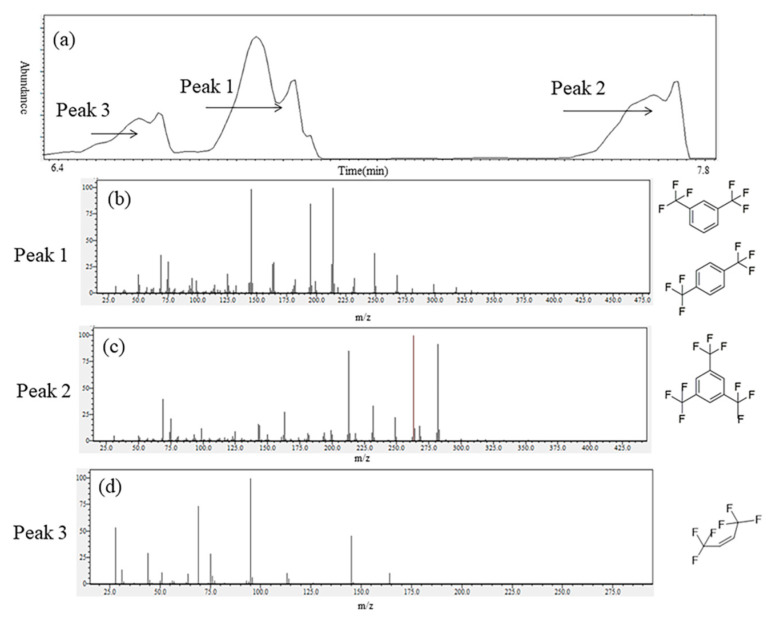
GC–MS results of oily liquids collected from the inner wall of the quartz tube. (**a**): Gas chromatogram of oily liquids products; (**b**): Mass spectrum of Peak 1; (**c**): Mass spectrum of Peak 2; (**d**): Mass spectrum of Peak 3.

As shown in Figure 4, flavescent oily liquids were always being observed on the inner wall of the quartz tube in our investigation. This phenomenon was consistent with a previous report [18]. Analysis of the products collected from the inner wall of the quartz tube was accomplished by GC–MS, and the results of GC–MS were shown in Figure 5. GC–MS temperature programming: initial oven temperature was 323 K; this was then raised to 513 K at 5 K/min and held for 5 min. Helium was used as carrier gas with a flow rate of 3 mL/min (constant flow). Programming temperature vaporizer (PTV) injector temperature was 513 K and the amount of injection was 1.0 μL at spilt mode (spilt ratio, 20:1). The MSD was operated in the electron impact (EI) mode. The ion source temperature was 503 K and the electron energy was 70 eV. The mass range from *m/z* 28 to 400 was scanned. Some compounds, such as 1,3,5-tris(trifluoromethyl)benzene, 1,4-bis(trifluoromethyl)benzene and 1,3-bis(trifluoromethyl)benzene, were existed and their possible structures are shown in Figure 5b,c. Among these components, 1,4-bis(trifluoromethyl)benzene and 1,3-bis(trifluoromethyl)benzene were isomers of each other. HFO-1336mzz (Z) was detected in flavescent oily liquids, and it is shown in Figure 5a,d.

#### 2.1.3. Coke Collected Form the Inner Wall of the Quartz Tube

As previously and widely described, the precursors of a series of large polycyclic compounds and coke could be small polycyclic compounds [30,31]. During HFO-1336mzz(Z) pyrolysis process, tiny carbon particles could be observed on the inner wall of the quartz tube over a short pyrolysis time, while a thin carbon layer could be observed over a long pyrolysis time.

**Figure 6:**
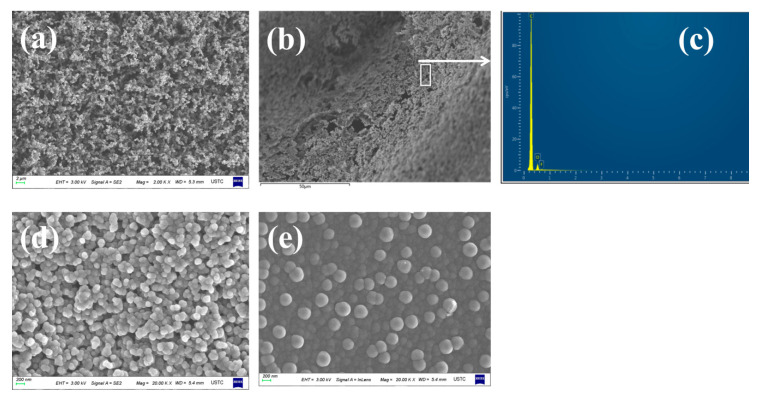
Carbon layer surface images at high magnification. ((**a**,**b**,**d**,**e**): SEM; (**c**): EDS spectrum)

The morphology of the coke collected from the inner wall of the quartz tube was characterized by scanning electronic microscopy (SEM) in Figure 6. As shown in Figure 6a, the thin carbon layer was piled up by many black carbon particles. Figure 6b,c showed the low magnification SEM image for the top-view surface of the thin carbon layer with energy dispersive spectroscopy (EDS) spectrum for C, O and F. The corresponding EDS spectrum in Figure 6c confirmed the thin carbon layer mainly composed by carbon element. The thin carbon layer contained trace amounts of oxygen and fluorine because of the products formed by the reaction of HF gas with the quartz wall during pyrolysis adhered to the coke. Both sides of the carbon layer surface images at high magnification are shown in Figure 6d,e. Carbon particles ranged in size from 50 nm to 200 nm. It was noted that carbon particles of a thin carbon layer in contact with HFO-1336mzz(Z) were growing freely, as shown in Figure 6d. Figure 6e illustrated that carbon particles in contact with the inner wall of the quartz tube were more closely, which is similar to the cell adhesion growth phenomenon. In the pyrolysis process, oily liquids would be further carbonized to form carbon spheres one-by-one and the carbon spheres were constantly attached to the wall of quartz tubes.

#### 2.1.4. The Trend of Hydrogen Fluoride Concentration

**Figure 7:**
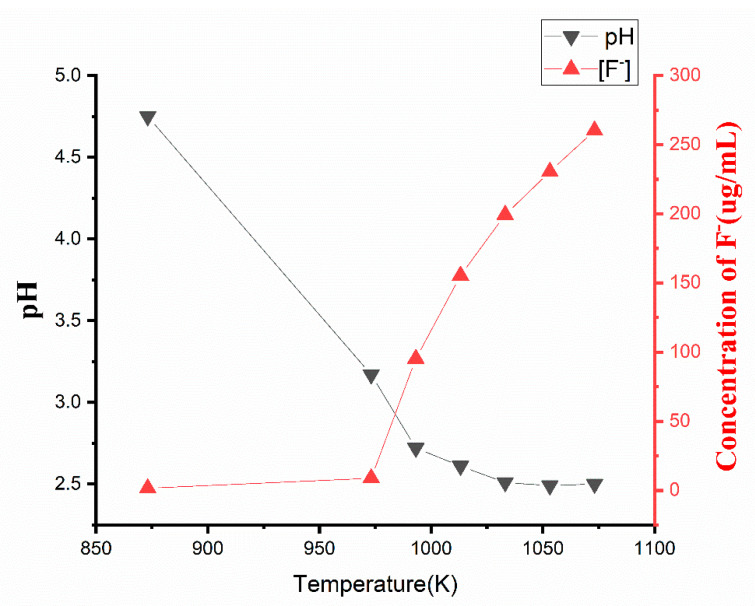
Relationships between pyrolysis temperature, pH and [F^−^].

Hydrogen fluoride (HF) is a very dangerous and toxic substance, and it not only corrodes facilities, but also damages human health. Thus, its variation trend with temperature in the entire HFO-1336mzz(Z) pyrolysis should be concerned. The results in Figure 7 indicated that [H^+^] and [F^−^] gradually increased with the increase of pyrolysis temperature (873 K to 1033 K). At 1033 K, [H^+^] remained still, while the concentration of fluoride ions significantly changed.

At 873 K, [H^+^] =10^−4.75^ mol L^−1^ and [F^−^] = 1.5252 μg mL^−1^ indicated that HFO-1336mzz(Z) slightly decomposed at this temperature. The changes in pH and fluoride ion concentration were remarkable at 993 K, which means that HF was produced in large quantities at this temperature. Because HF production remained unchanged at 1033 K, pH = 2.5 remained still. However, oily liquids products still increased, resulting in increasing the concentrations of fluoride ions significantly (1033 K to 1073 K). The production amount of hydrogen fluoride could be inferred from the content of hydrogen ions. According to the pH value of 400 mL deionized water, the flow rate of the peristaltic pump was set to 0.41 mL min^−1^, and the pyrolysis time was set to 30 min at 1033 K, it could be inferred that 1 L HFO-1336mzz(Z) (L) would produce 0.1mol of HF at least. However, we have also detected F in the experimental tube in Section 2.1.3, and HF were collected in the gas-collecting pocket. In other word, HF may react with the furnace and system walls on its way to the deionized water. Moreover, deionized water could not completely absorb the released HF. Therefore, the content of HF released in the experiment is higher than that detected indirectly.

### 2.2. Theoretical Calculation

#### 2.2.1. The Homolytic Cleavage Reactions of HFO-1336mzz(Z)

**Figure 8:**
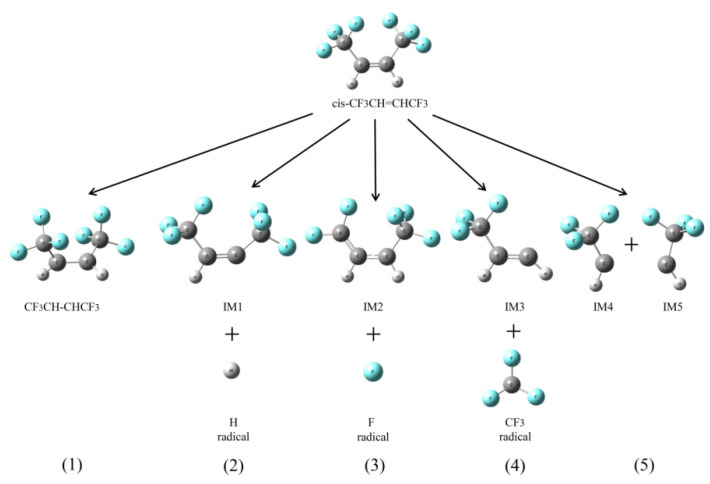
Main initiation reactions. C, H and F atoms are represented with black, gray and blue balls, respectively.

**Figure 9:**
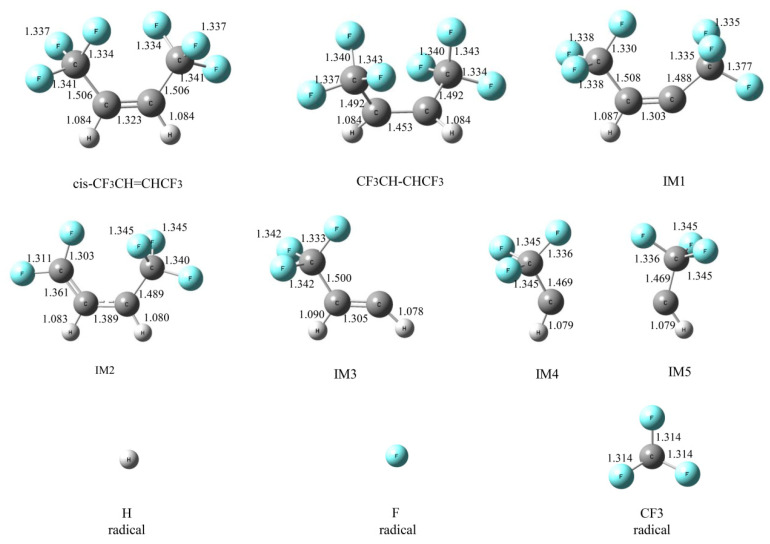
Geometric optimization of transition states, intermediates and radicals at the beginning of HFO-1336mzz pyrolysis (Z). C, H and F atoms are represented with black, gray and blue balls, respectively.

At the beginning of pyrolysis, *cis*-CF_3_CH=CHCF_3_ would produce three main radicals through homolytic cleavage reaction; its brief process is shown in Figure 8. Moreover, the HFO-1336mzz(Z) bond distance and bond dissociation energies (BEDs) are shown in Table 1. As shown in Figure 8 and Table 1, these three radicals including H, F and CF_3_ were generated through the homolytic cleavages of C–H, C–F and C–C bonds, and the respective energy barriers were 446.3, 444.0 and 427.5 kJ mol^−1^, respectively. Geometry optimization structures of *cis*-CF_3_CH=CHCF_3_, intermediates and radicals via homolytic cleavage reactions are shown in Figure 9. As shown in Figure 9 and Table 1, the lengths of C–H, C–F and C–C bonds from HFO-1336mzz(Z) molecular were 1.08, 1.34 and 1.51 Å, respectively. The longer the bond distance and the lower the BEDs, which means that the bond is easier to be broken. Therefore, the homolytic cleavage reaction of the C–C bond is easier to take place than the H-scission reaction and F-scission reaction, which are consistent with published literature [8]. It is worth noting that the energy barriers of homolytic cleavage of C–H and C–F bonds are extremely similar. However, the number of F atoms in the molecular structure was three times that of H atoms. Thus, the number of F radicals in this system may be more than that of H radicals at the beginning of HFO-1336mzz(Z) pyrolysis. Furthermore, the basic state *cis*-CF_3_CH=CHCF_3_ was excited into the lowest triplet state CF_3_CH–CHCF_3_, and the dissociation of the C=C bond was also taken into account in this work. It is worth noting that the dissociation of the C=C bond produced more energies than all other bonds in the HFO-1336mzz(Z) molecule (685.7 kJ mol^−1^), while the energy derived from activation of the basic state *cis*-CF_3_CH=CHCF_3_ was the lowest (240.7 kJ mol^−1^). This result was very consistent with some reports on the BTP pyrolysis [14] and HFO-1234yf pyrolysis mechanism [16].

As shown in Figure 8 and Figure 9, the *cis*-CF3CH=CHCF3 molecular structure was symmetrical, which means that two hydrogen atoms and six fluorine atoms are chemically equivalent. First, the basic state *cis*-CF_3_CH=CHCF_3_ was excited into the lowest triplet state CF_3_CH–CHCF_3_. Second, the H atom was related to the formation of H radical and IM1 and the F atom was associated with the formation of F radical and IM2. Moreover, the CF_3_ group was associated with the formation of CF_3_ radical and IM3. Finally, the C=C in *cis*-CF_3_CH=CHCF_3_ molecular was completely fractured to form IM4 and IM5, but which is very hard to take place due to the higher energy demand.

#### 2.2.2. Intramolecular Elimination

**Figure 10:**
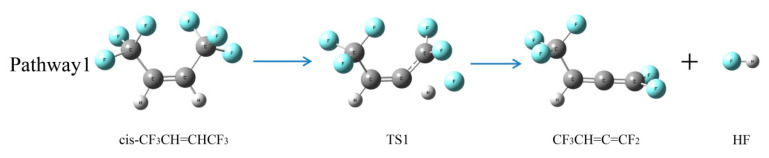
Intramolecular elimination of HFO-1336mzz(Z). C, H and F atoms are represented with black, gray and blue balls, respectively.

**Figure 11:**
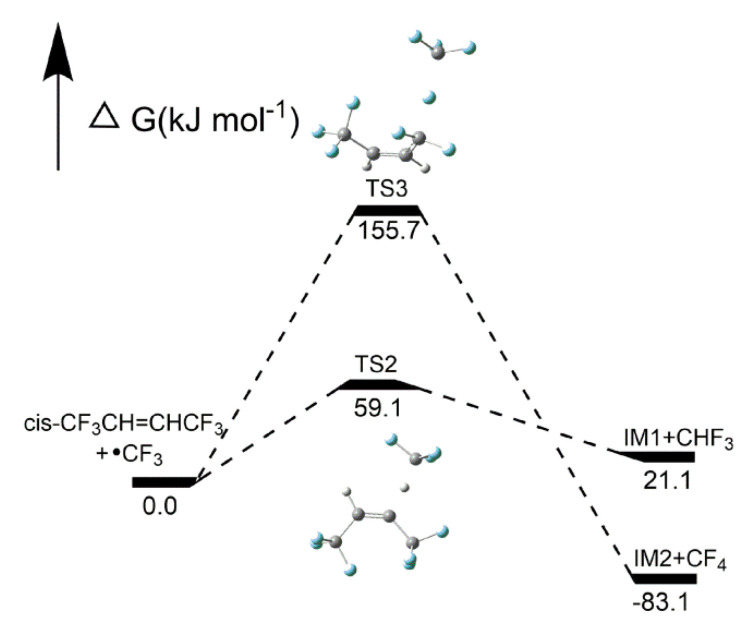
Energy profiles for intramolecular elimination reaction. C, H and F atoms are represented with black, gray and blue balls, respectively.

**Figure 12:**
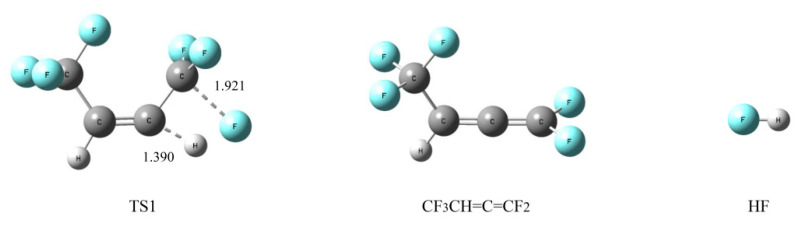
Geometric optimization of transition states, intermediates and radicals of intramolecular elimination reaction for HFO-1336mzz(Z). C, H and F atoms are represented with black, gray and blue balls, respectively.

As shown in Figure 10, the H atom and F atom on the two adjacent C atoms were separated from the respective C atom via transition state 1 (TS1) to form an HF molecule, and another product was CF_3_CH=C=CF. Another H atom of CF_3_CH=C=CF may be separated to form another HF molecule, which means that an HFO-1336mzz(Z) molecule may produce two HF molecules by an intramolecular elimination reaction. However, two C–C double bonds on the same C atom were considered to be unstable, so CF_3_CH=C=CF may be converted to other products. The more detailed energy profiles are shown in Figure 11, and the activation energy was 320.1 kJ mol^−1^. There were lower energy barriers in the intramolecular elimination reaction than those in the initial pyrolysis, which is consistent with our experimental results (Section 2.1.4) and the previous reports [18,20] demonstrating that intramolecular eliminations may be the most important pathway for HFO-1336mzz(Z) pyrolysis. As shown in Figure 12, some parameters in the elimination reaction showed the C–F bond length gradually increased from 1.341 Å to 1.921 Å (~43%) and the C–H bond length increased from 1.084 Å to 1.390 Å (~28%).

#### 2.2.3. H- and F-abstraction Reactions

HFO-1336mzz(Z) pyrolysis was caused by the radicals including H, F and CF_3_ produced at the beginning of thermal decomposition through chain reactions. Thanks to the activities of H, F and CF_3_ radicals, H- and F-abstraction reactions were supposed to the subsequent reactions.

##### Reaction pathways of *cis*-CF_3_CH=CHCF_3_ + CF_3_•

**Figure 13:**
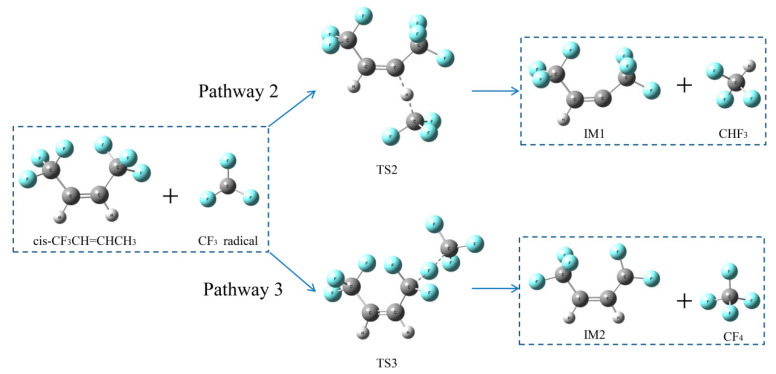
Two proposed pathways for *cis*-CF_3_CH=CHCF_3_+CF_3_• reactions. C, H and F atoms are represented with black, gray and blue balls, respectively.

**Figure 14:**
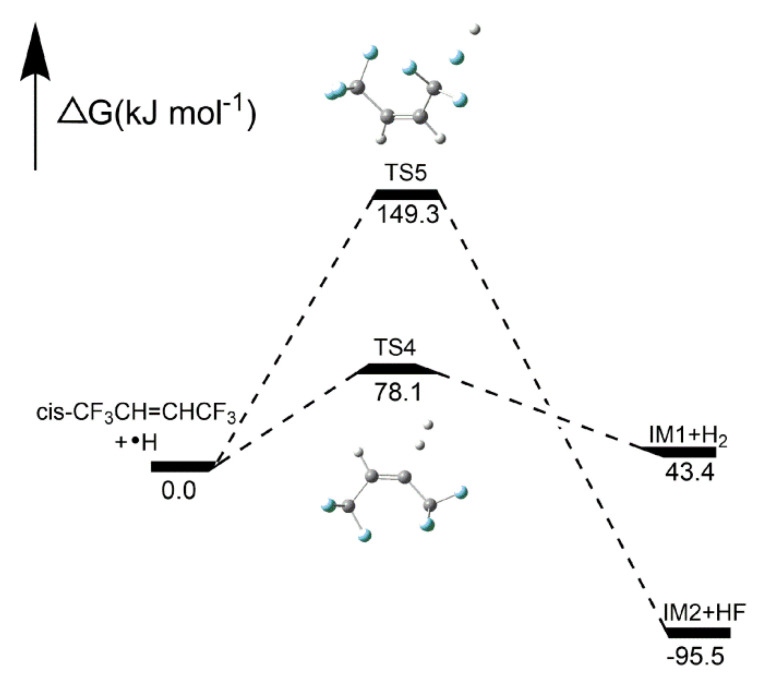
Energy profiles for reaction pathways of *cis*-CF_3_CH=CHCF_3_ + CF_3_•. C, H and F atoms are represented with black, gray and blue balls, respectively.

As shown in Figure 13, two pathways of *cis*-CF_3_CH=CHCF_3_ + CF_3_• reactions were demonstrated in this work. Pathway 2 was an H-abstraction reaction, which showed the H atom was replaced with a CF_3_ radical through TS2 to form CF_3_H and IM1. The F atom in *cis*-CF_3_CH=CHCF_3_ was abstracted via TS3 to form CF_4_ and IM2 through F-abstraction reactions (Pathway 3). As shown in Figure 14, the activation energies of pathways 2 and 3 were 59.1 kJ mol^−1^ and 155.7 kJ mol^−1^, respectively, which means that CF_3_H is easier to form than CF_4_.

##### Reaction pathways of *cis*-CF_3_CH=CHCF_3_ + H•

**Figure 15:**
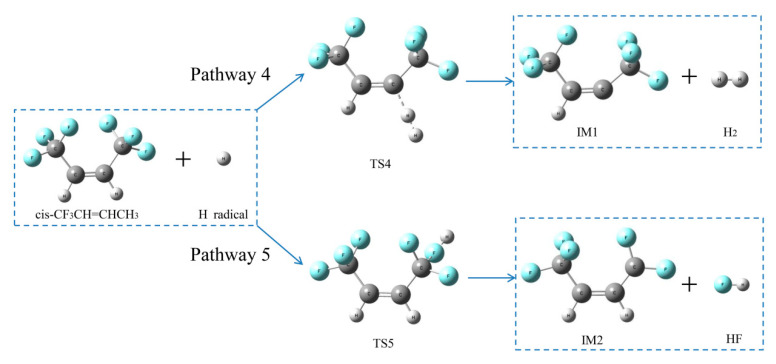
Two proposed pathways for *cis*-CF_3_CH=CHCF_3_ + H• reactions.

**Figure 16:**
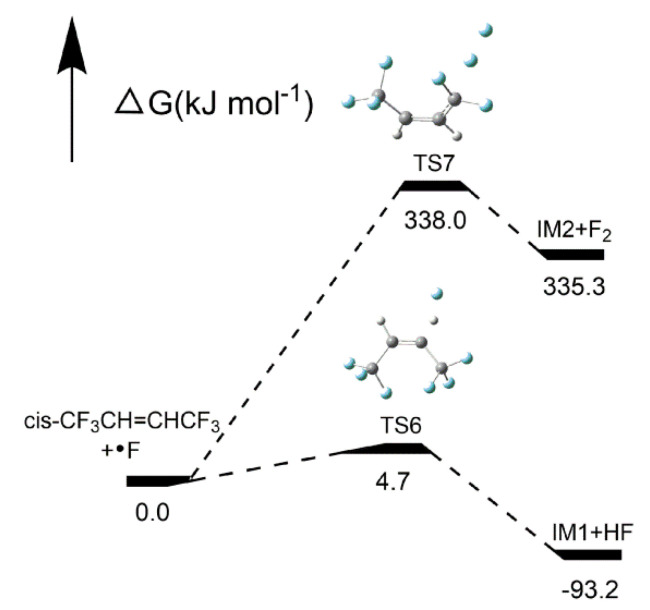
Energy profiles for reaction pathways of *cis*-CF_3_CH=CHCF_3_ + H•. C, H and F atoms are represented with black, gray and blue balls, respectively.

As shown in Figure 15, there were two pathways for the HFO-1336mzz(Z) reactions. Pathway 4 was an H-abstraction reaction, which indicates the H atom can be replaced with an H radical through TS4 to form H_2_ and IM1. F atom in *cis*-CF_3_CH=CHCF_3_ was abstracted via TS5 to form HF and IM2 through F-abstraction reactions (Pathway 5). As illustrated in Figure 16, the activation energies of pathways 4 and 5 were 78.1 and 149.3 kJ mol^−1^, respectively, which means H_2_ is easier to form than HF in *cis*-CF_3_CH=CHCF_3_ + H• reaction. However, H_2_ was also easy to react with carbon–carbon double bond at high temperature, so the amount of H_2_ in the system may be trace. The amount of F atom in the HFO-1336mzz(Z) molecule was three times as much as that of the H atom, so the probability of Pathway 5 was higher.

It can be found that the activation energy of Pathway 5 was almost half that of Pathway 1 (Intramolecular elimination reaction), and both two reactions may produce HF. The HF formed via F-abstraction reactions was substantially easier than that via intramolecular elimination reaction.

##### Reaction pathways of *cis*-CF_3_CH=CHCF_3_ + F•

**Figure 17:**
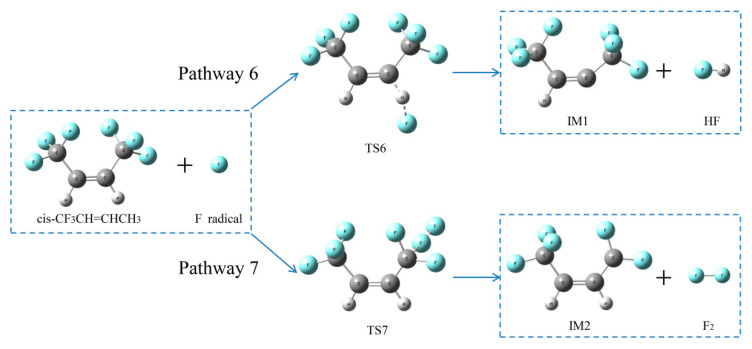
Two proposed pathways for *cis*-CF_3_CH=CHCF_3_ + F• reactions. C, H and F atoms are represented with black, gray and blue balls, respectively.

**Figure 18:**
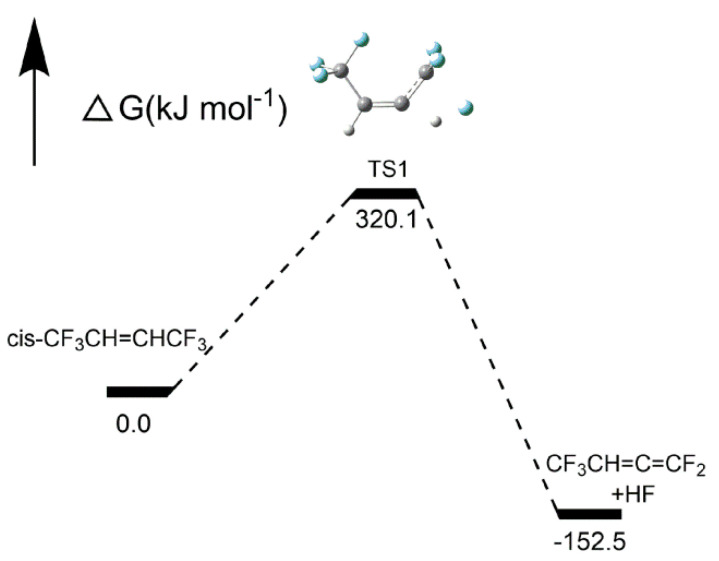
Energy profiles for reaction pathways of *cis*-CF_3_CH=CHCF_3_ + F•. C, H and F atoms are represented with black, gray and blue balls, respectively.

As shown in Figure 17, two possible pathways of *cis*-CF_3_CH=CHCF_3_ + F• were also calculated using the TS method. Pathway 6 was an H-abstraction reaction, which shows the H atom was abstracted by an H radical through TS6 to form HF and IM1. F atom in *cis*-CF_3_CH=CHCF_3_ was replaced through TS7 to form F_2_ and IM2 through F-abstraction reactions (Pathway 7). As shown in Figure 18, the activation energy of TS7 was only 4.7 kJ mol^−1^, which is lower than all other pathways (1 to 5). It also means that HF was extremely easy to form in the whole HFO-1336mzz(Z) pyrolysis process. The activation energy of TS6 was 338 kJ mol^−1^, which is the highest among all the mentioned pathways (pathways 1–5 and Pathway 7). These findings indicated that F_2_ was very hard to form in all intramolecular elimination reactions and H- and F-abstraction reactions.

#### 2.2.4. Formation Pathways of HF and Cyclic Products

According to the theoretical calculation results in Section 2.2.1 to 2.2.3, there were three possible pathways for the HFO-1336mzz(Z) dehydrofluorination: pathways 1, 5 and 6 (Table 2). Pathway 1 (320.1 kJ mol^−1^) was an intramolecular elimination reaction and was less endothermic, so it may be an important pathway for the initial decomposition of *cis*-CF_3_CHC=HCF_3_. Among other pathways, Pathway 6 (4.7 kJ mol^−1^) had much less activation energy than Pathway 7 (147.3 kJ mol^−1^), so the HF was easier to be generated through Pathway 6. According to the results of DFT calculation, the content of HF produced in the experiment is much lower.

**Figure 19:**
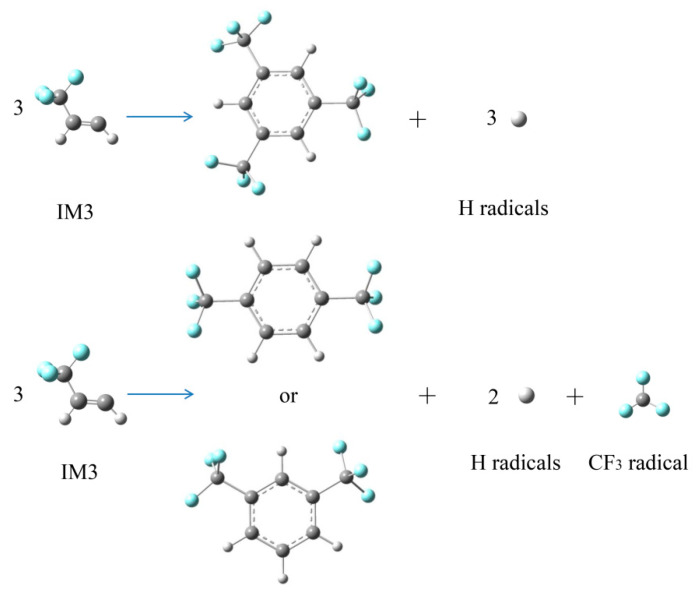
Possible reaction pathways of three polymers formation. C, H and F atoms are represented with black, gray and blue balls, respectively.

Based on the GC–MS results in Section 2.1.2, the relatively large molecular formation could be speculated as shown in Figure 19. In the initial reactions of HFO-1336mzz(Z), IM3 was generated through the homolytic cleavages of C–C bonds. With the increase of thermal decomposition temperature, IM3 could react further to form cyclic products.

## 3. Methodologies 

### 3.1. Materials

HFO-1336mzz(Z) (*cis*-CF_3_CH=CHCF_3_, purity ≥ 99.5%) was obtained from Kemu Fluoride Technology Co., Ltd., with no further purification before using. Ar is used in this thermal decomposition with experimental purity ≥ 99.99%. Deionized water and NaOH solution were prepared in the laboratory.

### 3.2. Experimental Equipment and Procedure

**Figure 20:**
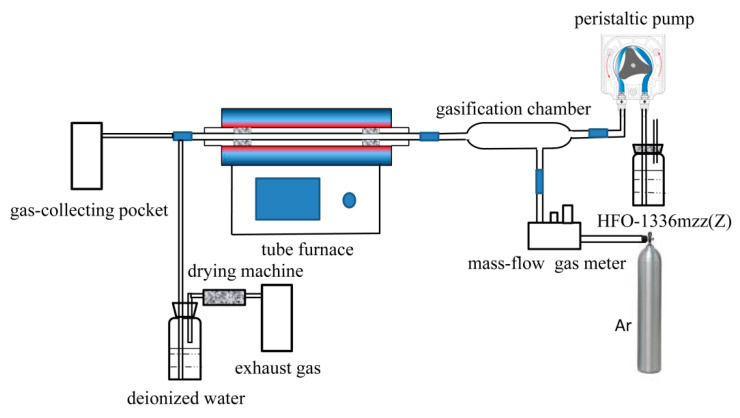
Schematic diagram for the HFO-1336mzz(Z) pyrolysis system.

According to the experimental system shown in Figure 20, the HFO-1336mzz(Z) pyrolysis process was studied. This thermal degradation device primarily includes three parts: (1) HFO-1336mzz(Z) storage and gasification unit, (2) reactor and (3) gas collecting and exhaust gas treating unit. The pure HFO-1336mzz(Z) (l) was preheated to 343 K in the gasification unit, and then it would be completely gasified.

Each quartz tube was first preheated to 1123 K for 1 h with Ar flow before the thermal degradation experiment. Then, the reactor was set to its designed temperature ranging from 873 K to 1073 K higher than previous studies [10], with a 225 mm constant temperature zone. The tubular furnace had a good temperature control system, and its reaction error was less than 5 K. The tubular furnace also exchanged quartz tubes to replace the new quartz tubes at different thermal decomposition temperatures. The inner diameter and length of the quartz tube were 10 mm and 1100 mm, respectively. During the experiment, the reaction temperatures of the tube furnace were set at 873 K, 973 K, 993 K, 1013 K, 1033 K, 1053 K and 1073 K, respectively. When the temperature reached the set value and held for 2 h, the heating device of the gasification unit was turned on and the flow rate of the peristaltic pump was 0.41 mL/min so that the liquid HFO-1336mzz at room temperature could be completely gasified in the gasification unit. The HFO-1336mzz(Z) residence time in constant temperature zone under at various was about 1–2 s. The pyrolysis duration of this experiment continued for 30 min. Gas-collecting pockets and deionized water collected the thermal decomposition products of HFO-1336mzz(Z) from the reactor. At the end of the thermal decomposition experiment, the exhaust gas needed to be treated with a sodium hydroxide solution before being discharged.

### 3.3. Theoretical Methodology

All calculations—including reactions and BDEs (bond dissociation energies)—were performed under the conditions of 873 K and 0.1 MPa, which was consistent with the initial conditions of the HFO-1336mzz(Z) pyrolysis system. The density functional M06-2X method [32,33] could optimize the reaction, products, and the transition states and has good accuracy for a fluorine-containing system. Hence, the M06-2X method was frequently used for analyzing and geometric optimization of HFO-1336mzz(Z) in this work. Moreover, intrinsic reaction coordinates (IRC) [34,35] calculations (static calculations) were conducted to confirm whether or not the identified transition states were related to the reactants and products. Gaussian 09W suit of programs is used to calculate all reactions [36].

### 3.4. GC–MS and IC Analysis

Gas chromatography and mass spectrometry (GC–MS) and IC (ion chromatography) were used to identify the pyrolysis products, including some small gas molecules and polycyclic compounds. GC–MS operation condition is shown in Table 3. The mass spectral identifications were carried out by comparing to the NIST14 and NIST14s. Hydrogen fluoride (HF) was absorbed by 400 mL deionized water for 30 min. Fluoride ion concentration and the pH of hydrofluoric acid solution were directly analyzed by Dionex ICS-3000 ion chromatography and acid and alkali meter, respectively.

## 4. Conclusions

In present study, the pyrolysis products of HFO-1336mzz(Z) were thoroughly studied from 873 to 1073 K. Furthermore, based on theoretical calculations, the generation pathways of HF and macromolecular products could be inferred.

(1) The initial pyrolysis temperature of HFO-1336mzz(Z) was about 873 K. When the reaction temperature reached at 973 K, the decomposition would become more intense and produced a white mist. The gas products turned into a yellow mist when the reaction temperature held at 993 K. In addition, coke was rapidly formed at 1073 K, which suggested that HFO-1336mzz(Z) not only performed simple pyrolysis, but also performed cyclization and polymerization reactions at high temperatures;

(2) The main thermal decomposition products are HF, the isomer of HFO-1336mzz(Z), macromolecular products and coke;

(3) The concentration of HF produced from the decomposition is increased with the increase of thermal decomposition temperature. Three pathways for HFO-1336mzz(Z) pyrolysis were explored to study the formation mechanism of HF through DFT simulations. Moreover, HF proved to generate through intramolecular elimination and abstraction reactions.

The results about HFO-1336mzz(Z) pyrolysis data and theoretical simulation could provide guidance and reference to HFO-1336mzz(Z) pyrolysis and toxicity assessment research.

## Figures and Tables

**Table 1:** Bond distances and Bond Dissociation Energies (BDEs) of different kinds of bonds.

Bonds	Bond Distance (Å)	Reaction Equations	BDEs (kJ · mol^−1^)	BDEs (298 K) (kJ · mol^−1^) [11]
C–H	1.08	CF_3_CH=CHCF_3_→CF_3_CH(C·)CF_3_ + H (2)	446.3	460.8
C–F	1.34	CF_3_CH=CHCF_3_→CF_3_CHCH(C·)CF_2_ + F (3)	444.0	454.5
C–C	1.51	CF_3_CH=CHCF_3_→CF_3_CHCH(C·) + CF_3_ (4)	427.5	431.2
C=C	1.32	CF_3_CH=CHCF_3_→CF_3_(C·)H(C·)HCF_3_ (1)	240.7	238.6
		CF_3_CH=CHCF_3_→2CF_3_(C·)H (5)	685.7	

**Table 2:** Typical pathways of hydrogen fluoride (HF) formation and energy barrier.

Pathway	Reaction	Energy Barrier (kJ · mol^−1^)
Pathway 1	*cis*-CF_3_CH=CHCF_3_→CF_3_CH=C=CF_2_ + HF	320.1
Pathway 5	*cis*-CF_3_CH=CHCF_3_+H•→IM2 + HF	4.7
Pathway 6	*cis*-CF_3_CH=CHCF_3_+F•→IM1 + HF	147.3

**Table 3:** GC–MS operation condition.

Parameters	GC–MS
Instrument	Shimadzu GCMS-QP2010 Plus, Ultra
Column	BD-624,30.0 m, 0.25 mm, 1.4 μm BD-5,60.0 m, 0.25 mm, 1.4 μm
Carrier gas flow rate	3.0 mL/min
Column temperature	323 K
Split ratio	1:20
Injection temperature	513 K
Ion source temperature	503 K
Ionization method	Electron impact
Ionization energy	70 eV
Scanning range	*m/z* 28.0–400
